# Bathing a Sanitary Need

**Published:** 1891-05

**Authors:** 


### BATHING A SANITARY NEED.

We clip the following excellent article from the *Healthy Home:*

The skin discharges the most important functions. Therefore personal cleanliness cannot be systematically neglected without risk to health. The quantity of water excreted by the skin is on the average about double that given off by the lungs in the same time, and in addition to water, carbonic acid gas and other used up products are constantly thrown off by the skin.

Then again the flattened cells or scales of the scarf or extreme outside skin are being constantly cast off, but by contact with the clothing and mixed with oily secretions of the skin they form a thin crust, as it were, which covers the whole body. This attracts the floating dust, ever present in the atmosphere, and the result is that healthy life is disturbed, more than a proper share of work is thrown on the lungs, kidneys and other eliminating organs, the blood is not properly purified and disorders of the skin are induced. The whole surface of the body from head to foot should be cleansed, at least once a day. Neglect of personal cleanliness is by no means confined to the poorer classes. There are numbers of well-to-do people who seldom or never wash, and to whom the "morning tub" is an unknown auxiliary to health and comfort. Yet it takes very little time, expense or trouble to secure ablution of the body of some sort. A hand-basin, a sponge, a shallow bath or flat tub, a piece of good white soap with no excess of alkali, a couple of gallons of water, and a towel, are all that are required; and the whole process need not occupy more than five minutes. Even rubbing the body first with a dry towel will, in most cases, keep the skin sufficiently clean during the week and promote healthy reaction, provided a warm bath, with a good soaping, is taken at the close of the week. A good flesh brush is also a valuable adjunct.

Infants should always be bathed in warm water, and if cold water is ever used the transition should be very gradual, and only tried in the summer time. Indeed, there is no doubt that a great many children contract a strong aversion to cold water, because they have injudiciously and thoughtlessly been subjected to the cold bath at periods when they were unable to bear it. A convenient time for taking a bath is just after getting out of bed in the morning or just before going to bed. This latter time is appropriate, as a bath is found to be very refreshing after severe or prolonged exercise of any kind.

In drying the skin, various kinds of towels may be used, but the effects produced by friction will be all the more beneficial if the skin can tolerate a rough towel. Flesh-gloves or hair-gloves can also be used to advantage.

As to the time spent in bathing, much depends on the kind of bath and the condition and constitution of the bather. Three minutes is quite sufficient for the cold sponge bath or cold plunge. In the tepid or warm bath the period may be prolonged to ten minutes or a quarter of an hour.

